# (*R*,*R*)-1-Acetyl-1′-(2,4,6-trinitro­phen­yl)-2,2′-bipyrrolidine

**DOI:** 10.1107/S1600536812051161

**Published:** 2012-12-22

**Authors:** Katarzyna Eichstaedt, Teresa Olszewska, Maria Gdaniec

**Affiliations:** a Department of Organic Chemistry, Gdańsk University of Technology, 80-233 Gdańsk, Poland; b Faculty of Chemistry, Adam Mickiewicz University, 60-780 Poznań, Poland

## Abstract

The structure of the title mol­ecule, C_16_H_19_N_5_O_7_, is mainly determined by the steric effect of a bulky 2,4,6-trinitro­phenyl group attached to the N atom of a pyrrolidine ring. Both pyrrolidine rings adopt an envelope conformation, with one of the methylene C atoms as the flap in each case, and the N—C—C—N torsion angle along the bond connecting the two pyrrolidine rings is −174.9 (2)°. The benzene ring of the 2,3,5-trinitro­phenyl substituent is deformed and the r.m.s. deviation of its six atoms from the best plane is 0.026 Å. The N atoms of the two nitro groups in the *ortho* positions deviate from the best plane of the benzene ring by −0.033 (5) and 0.385 (5) Å. These groups, as well as the pyrrolidine ring, are twisted relative to the aromatic ring in the same direction, their best planes forming dihedral angles of 30.2 (2), 64.8 (1) and 46.6 (2)°, respectively, with the ring. An intra­molecular C—H⋯O hydrogen bond occurs. In the crystal, there is a short [O⋯C = 3.019 (4) Å] contact between a nitro O atom and a C atom of the benzene ring bearing the nitro group and a C—H⋯O inter­action between a methyl H atom and another nitro O atom.

## Related literature

For crystal structures of related 1-amino-2,4,6-trinitro­benzenes, see: Butcher *et al.* (1992 ▶); Baggio *et al.* (1997 ▶).
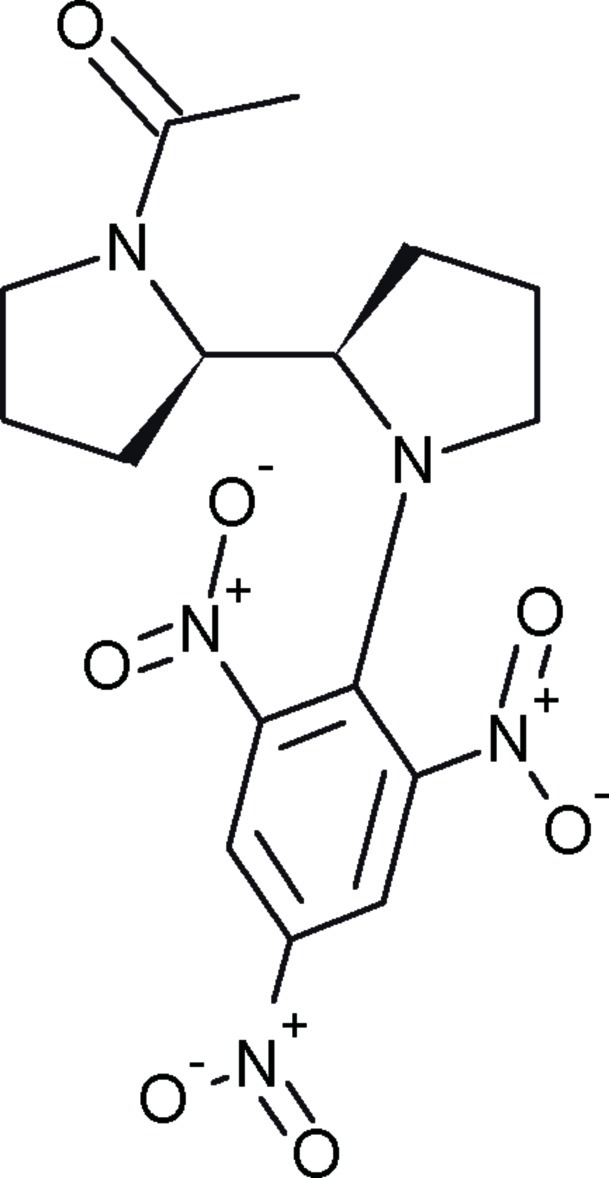


## Experimental

### 

#### Crystal data

C_16_H_19_N_5_O_7_
*M* *_r_* = 393.36
Orthorhombic, 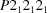

*a* = 8.1989 (5) Å
*b* = 10.4442 (6) Å
*c* = 20.8877 (13) Å
*V* = 1788.63 (19) Å^3^
*Z* = 4
Mo *K*α radiation
μ = 0.12 mm^−1^
*T* = 293 K
0.20 × 0.20 × 0.15 mm

#### Data collection

Oxford Diffraction Xcalibur Eos diffractometer
Absorption correction: multi-scan (*CrysAlis PRO*; Agilent, 2012 ▶) *T*_min_ = 0.990, *T*_max_ = 1.000
7678 measured reflections
1818 independent reflections
1477 reflections with *I* > 2σ(*I*)
*R*_int_ = 0.040

#### Refinement

*R*[*F* ^2^ > 2σ(*F* ^2^)] = 0.043
*wR*(*F* ^2^) = 0.090
*S* = 1.06
1818 reflections
254 parameters
H-atom parameters constrained
Δρ_max_ = 0.14 e Å^−3^
Δρ_min_ = −0.16 e Å^−3^

### 

Data collection: *CrysAlis PRO* (Agilent, 2012 ▶); cell refinement: *CrysAlis PRO*; data reduction: *CrysAlis PRO*; program(s) used to solve structure: *SHELXS97* (Sheldrick, 2008 ▶); program(s) used to refine structure: *SHELXL97* (Sheldrick, 2008 ▶); molecular graphics: *ORTEP-3 for Windows* (Farrugia, 1997 ▶) and *Mercury* (Macrae *et al.*, 2006 ▶); software used to prepare material for publication: *SHELXL97*.

## Supplementary Material

Crystal structure: contains datablock(s) global, I. DOI: 10.1107/S1600536812051161/rz5033sup1.cif

Structure factors: contains datablock(s) I. DOI: 10.1107/S1600536812051161/rz5033Isup2.hkl

Supplementary material file. DOI: 10.1107/S1600536812051161/rz5033Isup3.cml

Additional supplementary materials:  crystallographic information; 3D view; checkCIF report

## Figures and Tables

**Table 1:** Hydrogen-bond geometry (Å, °)

*D*—H⋯*A*	*D*—H	H⋯*A*	*D*⋯*A*	*D*—H⋯*A*
C2—H2⋯O1	0.98	2.18	2.891 (4)	129
C18—H18*C*⋯O2^i^	0.96	2.51	3.454 (5)	168

Symmetry code: (i) 
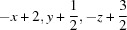
.

**Fig. 1.**
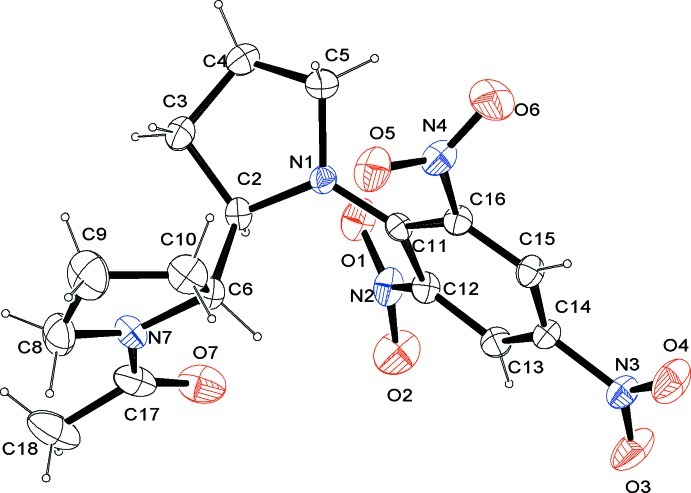
The molecular structure of the title compound with displacement ellipsoids shown at the 50% probability level.

**Fig. 2.**
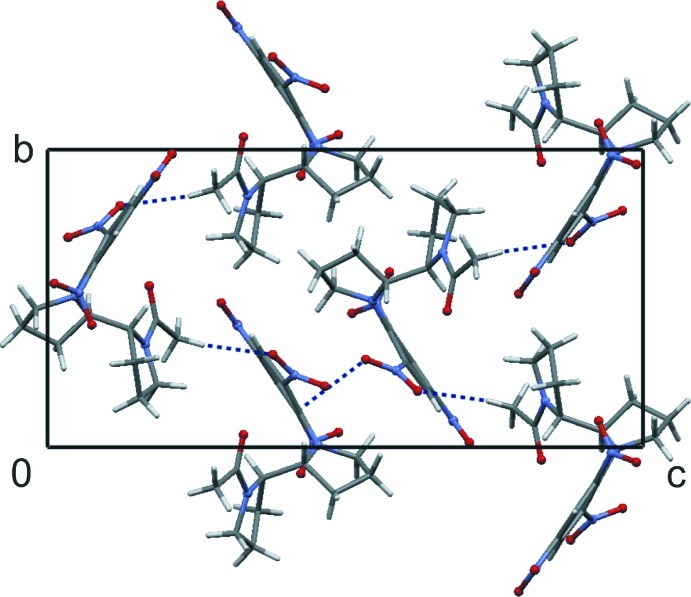
Crystal packing shown along the a axis. Short intermolecular contacts are shown as dashed lines.

## supplementary crystallographic information

### Comment

The title compound was synthesized as a part of a project aiming at the application of 2,4,6-trinitrophenyl chromophore for determination of absolute configuration of secondary diamines. The molecular structure of the title compound is shown in Fig. 1. Both pyrrolidine rings adopt an envelope conformation with the methylene C4 and C9 atoms forming a flap in each of the five-membered rings, respectively. The N7—C6—C2—N1 torsion angle along the bond connecting two pyrrolidine rings is -174.9 (2)°.

The benzene ring of the 2,3,5-trinitrophenyl substituent shows large deformation from planarity with r.m.s. deviation of 0.026 Å for the six fitted atoms and the maximum deviation from the best plane of 0.038 (2) Å for C11. Whereas N3 and N4 atoms of the nitro groups are vitrually in the mean plane of the benzene ring [their deviations from the plane being -0.050 (5), -0.033 (5) Å, respectively] the N1 atom from the pyrrolidine substituent and the N2 atom from one of the *ortho* nitro groups deviate strongly from this plane [deviations of -0.168 (4) and 0.385 (5) Å, respectively] reflecting steric effects within this overcrowded molecule. The nitro groups attached to C12 and C16 of the benzene ring are twisted in the same direction as the pyrrolidine ring attached to C11 forming the fragment of a propeller. The dihedral angles formed by these nitro groups and the planar C11, N1, C2, C5 fragment are 30.2 (2), 64.8 (1) 46.6 (2)°, respectively. The nitro group attached to C14 is only slightly twisted relative to the benzene ring with the dihedral angle of 4.9 (2)°. The conformation adopted by the molecule leads to two short intermolecular contacts between the pyrrolidine ring H atoms and O toms of the *ortho* nitro-groups (Table 1). Interestingly, the release of strain in the title molecule occurs differently than in 1-pyrrolidino-2,4,6-trinitrobenzene (Baggio *et al.*, 1997) where the benzene ring adopted a sofa form with the flap formed by the C atom to which the pyrrolidine ring was attached. On the other hand, the release of strain is similar to that observed for *N*,*N*-dimethyl-2,4,6-trinitroaniline (Butcher *et al.*, 1992), 1-piperidylo-2,4,6-trinitrobenzene and 1-morpholino-2,4,6-trinitrobenzene (Baggio *et al.*, 1997)

Two short intermolecular contacts are observed in this crystal structure. One, O1···.C16(1/2 + *x*, 3/2 - *y*, 2 - *z*) of 3.019 (4) Å, is formed between the nitro group O atom and the carbon atom of the benzene ring bearing the nitro group. The second one, H18C···.O2(2 - *x*, 1/2 + *y*, 3/2 - *z*) of 2.51 Å, is formed between the methyl group H atom and the nitro group O atom. The crystal packing in the studied crystal is shown in Fig. 2.

### Experimental

A mixture of (*R,R)*-2,2'-bipyrrolidine hydrochloride (280 mg, 1.31 mmol), 1-chloro-2,4,6-trinitrobenzene (650 mg, 2.63 mmol) and anhydrous sodium acetate (860 mg, 10.50 mmol) in anhydrous ethanol (10 ml) was heated under reflux for 30 min. The resulting suspension was cooled to room temperature and water (15 ml) was added into it. The aqueous layer was extracted with dichloromethane (2 *x* 15 ml). The combined organic extracts were dried over anhydrous magnesium sulfate. Filtration of the drying agent and removal the solvent *in vacuo* afforded the crude product, which was purified by means of column chromatography on silica gel using ethyl acetate to yield 0.12 g (23%) of product as a yellow solid. Crystals suitable for X-ray diffraction analysis were obtained by allowing a refluxed solution of the product in ethyl acetate to cool slowly at room temperature (without temperature control) and allowing the solvent to evaporate for 20 h, ^1^H NMR: (CDCl_3_, 500 MHz) δ: 8.66 (s, 2H); 4.49 (q, *J*=6.8 Hz, 1H); 4.09 (q, *J*=6.0 Hz, 1H); 3.56 (m, 1H); 3.43 (m, 1H); 3.36 (m, 1H); 3.20 (t, *J*=8.3 Hz, 1H); 2.10 (m, 2H); 1.92 (s, 3H); 1,88 (m, 5H); 1.73 (m, 1H), [α]_D_^20^ = -1430 (c 0.2 e thyl acetate).

### Refinement

All H atoms were located in electron-density difference maps, however for further refinement their positions were determined geometrically with C—H bond lengths of 0.93 - 0.97 Å. All H atoms were refined in the riding-model approximation, with *U*_iso_(*H*)=1.5*U*_eq_(C_methyl_) or *U*_iso_(H)=1.2*U*_eq_(C) for the remaining H atoms. In the absence of significant anomalous dispersion effects, 1319 Friedel pairs were merged.

### Crystal data

**Table:**

C_16_H_19_N_5_O_7_	*F*(000) = 824
*M**_r_* = 393.36	*D*_x_ = 1.461 Mg m^−^^3^
Orthorhombic, *P*2_1_2_1_2_1_	Mo *K*α radiation, λ = 0.71073 Å
Hall symbol: P 2ac 2ab	Cell parameters from 1951 reflections
*a* = 8.1989 (5) Å	θ = 2.9–28.7°
*b* = 10.4442 (6) Å	µ = 0.12 mm^−^^1^
*c* = 20.8877 (13) Å	*T* = 293 K
*V* = 1788.63 (19) Å^3^	Tabloid, orange
*Z* = 4	0.20 × 0.20 × 0.15 mm

### Data collection

**Table:**

Oxford Diffraction Xcalibur Eos diffractometer	1818 independent reflections
Radiation source: Enhance (Mo) X-ray Source	1477 reflections with *I* > 2σ(*I*)
Graphite monochromator	*R*_int_ = 0.040
Detector resolution: 16.1544 pixels mm^-1^	θ_max_ = 25.0°, θ_min_ = 4.3°
ω scan	*h* = −9→9
Absorption correction: multi-scan (*CrysAlis PRO*; Agilent, 2012)	*k* = −12→12
*T*_min_ = 0.990, *T*_max_ = 1.000	*l* = −24→24
7678 measured reflections	

### Refinement

**Table:**

Refinement on *F*^2^	Primary atom site location: structure-invariant direct methods
Least-squares matrix: full	Secondary atom site location: difference Fourier map
*R*[*F*^2^ > 2σ(*F*^2^)] = 0.043	Hydrogen site location: inferred from neighbouring sites
*wR*(*F*^2^) = 0.090	H-atom parameters constrained
*S* = 1.06	*w* = 1/[σ^2^(*F*_o_^2^) + (0.0351*P*)^2^ + 0.2637*P*] where *P* = (*F*_o_^2^ + 2*F*_c_^2^)/3
1818 reflections	(Δ/σ)_max_ = 0.001
254 parameters	Δρ_max_ = 0.14 e Å^−^^3^
0 restraints	Δρ_min_ = −0.16 e Å^−^^3^

### Special details

**Table:**

Geometry. All e.s.d.'s (except the e.s.d. in the dihedral angle between two l.s. planes) are estimated using the full covariance matrix. The cell e.s.d.'s are taken into account individually in the estimation of e.s.d.'s in distances, angles and torsion angles; correlations between e.s.d.'s in cell parameters are only used when they are defined by crystal symmetry. An approximate (isotropic) treatment of cell e.s.d.'s is used for estimating e.s.d.'s involving l.s. planes.
Refinement. Refinement of *F*^2^ against ALL reflections. The weighted *R*-factor *wR* and goodness of fit *S* are based on *F*^2^, conventional *R*-factors *R* are based on *F*, with *F* set to zero for negative *F*^2^. The threshold expression of *F*^2^ > σ(*F*^2^) is used only for calculating *R*-factors(gt) *etc*. and is not relevant to the choice of reflections for refinement. *R*-factors based on *F*^2^ are statistically about twice as large as those based on *F*, and *R*- factors based on ALL data will be even larger.

### Fractional atomic coordinates and isotropic or equivalent isotropic displacement parameters (Å^2^)

**Table:**

	*x*	*y*	*z*	*U*_iso_*/*U*_eq_	
N1	0.6350 (3)	0.9926 (2)	0.95416 (11)	0.0336 (6)	
C2	0.7856 (4)	1.0547 (3)	0.93068 (14)	0.0360 (8)	
H2	0.8740	0.9915	0.9309	0.043*	
C3	0.8200 (5)	1.1543 (3)	0.98208 (15)	0.0496 (10)	
H3A	0.9361	1.1704	0.9860	0.060*	
H3B	0.7647	1.2343	0.9727	0.060*	
C4	0.7528 (5)	1.0932 (4)	1.04212 (16)	0.0520 (10)	
H4A	0.7335	1.1566	1.0752	0.062*	
H4B	0.8264	1.0283	1.0585	0.062*	
C5	0.5943 (5)	1.0343 (4)	1.01938 (16)	0.0498 (10)	
H5A	0.5628	0.9623	1.0460	0.060*	
H5B	0.5068	1.0968	1.0191	0.060*	
C6	0.7620 (4)	1.1031 (3)	0.86178 (15)	0.0378 (8)	
H6	0.7269	1.0313	0.8349	0.045*	
N7	0.9150 (4)	1.1543 (3)	0.83607 (13)	0.0429 (7)	
C8	0.9020 (6)	1.2884 (4)	0.81637 (19)	0.0625 (12)	
H8A	0.8998	1.2957	0.7701	0.075*	
H8B	0.9928	1.3381	0.8328	0.075*	
C9	0.7443 (6)	1.3326 (4)	0.8449 (2)	0.0804 (15)	
H9A	0.6904	1.3929	0.8166	0.097*	
H9B	0.7632	1.3740	0.8859	0.097*	
C10	0.6410 (5)	1.2131 (4)	0.85355 (18)	0.0562 (10)	
H10A	0.5726	1.1991	0.8163	0.067*	
H10B	0.5719	1.2209	0.8910	0.067*	
C11	0.5621 (4)	0.8893 (3)	0.92546 (13)	0.0300 (7)	
C12	0.6456 (4)	0.7851 (3)	0.89814 (14)	0.0331 (7)	
C13	0.5699 (4)	0.6911 (3)	0.86275 (15)	0.0362 (8)	
H13	0.6310	0.6270	0.8432	0.043*	
C14	0.4047 (4)	0.6931 (3)	0.85668 (14)	0.0330 (7)	
C15	0.3111 (4)	0.7849 (3)	0.88681 (14)	0.0350 (8)	
H15	0.1978	0.7823	0.8849	0.042*	
C16	0.3898 (4)	0.8793 (3)	0.91946 (14)	0.0332 (8)	
C17	1.0360 (5)	1.0730 (5)	0.81772 (16)	0.0562 (11)	
C18	1.1766 (6)	1.1325 (5)	0.78229 (19)	0.0863 (16)	
H18A	1.2647	1.0721	0.7796	0.129*	
H18B	1.2127	1.2076	0.8047	0.129*	
H18C	1.1423	1.1557	0.7399	0.129*	
O1	0.8695 (3)	0.7949 (2)	0.96552 (12)	0.0539 (7)	
O2	0.8947 (3)	0.6887 (3)	0.87822 (14)	0.0715 (9)	
O3	0.4126 (4)	0.5153 (3)	0.79145 (14)	0.0718 (9)	
O4	0.1779 (3)	0.5873 (3)	0.81795 (14)	0.0696 (9)	
O5	0.3003 (4)	1.0886 (2)	0.92558 (14)	0.0613 (8)	
O6	0.1894 (3)	0.9500 (3)	0.98862 (14)	0.0702 (9)	
O7	1.0284 (4)	0.9579 (3)	0.82913 (13)	0.0685 (8)	
N2	0.8176 (4)	0.7573 (3)	0.91450 (16)	0.0424 (7)	
N3	0.3249 (4)	0.5912 (3)	0.81954 (13)	0.0425 (7)	
N4	0.2857 (4)	0.9810 (3)	0.94709 (15)	0.0451 (7)	

### Atomic displacement parameters (Å^2^)

**Table:**

	*U*^11^	*U*^22^	*U*^33^	*U*^12^	*U*^13^	*U*^23^
N1	0.0302 (16)	0.0338 (14)	0.0369 (14)	−0.0035 (12)	0.0035 (13)	−0.0049 (13)
C2	0.0333 (19)	0.0329 (17)	0.0416 (18)	−0.0042 (15)	−0.0008 (16)	0.0035 (15)
C3	0.056 (3)	0.047 (2)	0.0456 (19)	−0.0165 (19)	−0.0053 (19)	−0.0037 (18)
C4	0.064 (3)	0.051 (2)	0.0407 (19)	−0.011 (2)	−0.0036 (19)	−0.0070 (18)
C5	0.056 (2)	0.049 (2)	0.0438 (19)	−0.007 (2)	0.0119 (19)	−0.0098 (17)
C6	0.039 (2)	0.0334 (16)	0.0414 (17)	−0.0030 (16)	−0.0034 (16)	0.0017 (16)
N7	0.0406 (19)	0.0458 (16)	0.0425 (15)	0.0007 (15)	0.0052 (14)	0.0095 (14)
C8	0.076 (3)	0.045 (2)	0.066 (3)	−0.011 (2)	−0.005 (2)	0.015 (2)
C9	0.099 (4)	0.046 (2)	0.097 (3)	0.011 (3)	0.004 (3)	0.021 (2)
C10	0.051 (3)	0.060 (2)	0.058 (2)	0.012 (2)	−0.006 (2)	0.010 (2)
C11	0.0288 (19)	0.0297 (16)	0.0316 (15)	0.0013 (15)	0.0019 (14)	0.0035 (14)
C12	0.0230 (18)	0.0348 (17)	0.0413 (17)	0.0034 (15)	−0.0018 (15)	0.0006 (16)
C13	0.036 (2)	0.0318 (17)	0.0408 (17)	0.0057 (16)	0.0029 (16)	−0.0019 (16)
C14	0.0320 (19)	0.0310 (17)	0.0358 (16)	0.0000 (15)	−0.0035 (15)	−0.0045 (15)
C15	0.0255 (18)	0.0355 (17)	0.0441 (17)	−0.0003 (16)	−0.0036 (15)	−0.0027 (16)
C16	0.0283 (19)	0.0307 (17)	0.0407 (17)	0.0052 (15)	0.0022 (15)	−0.0035 (15)
C17	0.047 (2)	0.086 (3)	0.036 (2)	0.006 (2)	0.0034 (19)	0.005 (2)
C18	0.055 (3)	0.147 (5)	0.056 (2)	0.006 (3)	0.017 (2)	0.016 (3)
O1	0.0466 (17)	0.0480 (15)	0.0671 (16)	0.0003 (14)	−0.0241 (14)	0.0041 (14)
O2	0.0383 (16)	0.080 (2)	0.096 (2)	0.0216 (16)	0.0010 (16)	−0.0242 (18)
O3	0.0569 (19)	0.0639 (17)	0.094 (2)	0.0079 (16)	−0.0072 (16)	−0.0447 (17)
O4	0.0368 (17)	0.0778 (19)	0.094 (2)	−0.0062 (16)	−0.0110 (16)	−0.0304 (17)
O5	0.0602 (18)	0.0344 (14)	0.089 (2)	0.0120 (13)	−0.0011 (16)	−0.0061 (14)
O6	0.0522 (18)	0.075 (2)	0.0832 (19)	0.0180 (16)	0.0279 (17)	−0.0056 (16)
O7	0.075 (2)	0.0656 (19)	0.0646 (16)	0.0316 (17)	0.0110 (16)	0.0027 (15)
N2	0.0293 (17)	0.0349 (16)	0.0631 (19)	0.0033 (13)	−0.0052 (16)	−0.0001 (15)
N3	0.0393 (19)	0.0414 (16)	0.0468 (17)	−0.0003 (16)	−0.0061 (15)	−0.0078 (15)
N4	0.0324 (18)	0.0457 (18)	0.0572 (18)	0.0083 (15)	−0.0044 (16)	−0.0130 (17)

### Geometric parameters (Å, º)

**Table:**

N1—C11	1.371 (4)	C10—H10A	0.9700
N1—C5	1.469 (4)	C10—H10B	0.9700
N1—C2	1.479 (4)	C11—C12	1.407 (4)
C2—C3	1.522 (4)	C11—C16	1.422 (4)
C2—C6	1.538 (4)	C12—C13	1.376 (5)
C2—H2	0.9800	C12—N2	1.479 (4)
C3—C4	1.512 (5)	C13—C14	1.361 (4)
C3—H3A	0.9700	C13—H13	0.9300
C3—H3B	0.9700	C14—C15	1.380 (4)
C4—C5	1.514 (5)	C14—N3	1.471 (4)
C4—H4A	0.9700	C15—C16	1.362 (4)
C4—H4B	0.9700	C15—H15	0.9300
C5—H5A	0.9700	C16—N4	1.479 (4)
C5—H5B	0.9700	C17—O7	1.227 (5)
C6—N7	1.466 (4)	C17—C18	1.504 (6)
C6—C10	1.527 (5)	C18—H18A	0.9600
C6—H6	0.9800	C18—H18B	0.9600
N7—C17	1.361 (5)	C18—H18C	0.9600
N7—C8	1.463 (4)	O1—N2	1.213 (4)
C8—C9	1.497 (6)	O2—N2	1.220 (4)
C8—H8A	0.9700	O3—N3	1.220 (4)
C8—H8B	0.9700	O4—N3	1.206 (4)
C9—C10	1.519 (6)	O5—N4	1.216 (4)
C9—H9A	0.9700	O6—N4	1.217 (4)
C9—H9B	0.9700		
			
C11—N1—C5	122.7 (3)	C8—C9—H9B	110.5
C11—N1—C2	124.3 (2)	C10—C9—H9B	110.5
C5—N1—C2	111.6 (2)	H9A—C9—H9B	108.7
N1—C2—C3	102.8 (3)	C9—C10—C6	105.6 (3)
N1—C2—C6	110.5 (3)	C9—C10—H10A	110.6
C3—C2—C6	117.3 (3)	C6—C10—H10A	110.6
N1—C2—H2	108.7	C9—C10—H10B	110.6
C3—C2—H2	108.7	C6—C10—H10B	110.6
C6—C2—H2	108.7	H10A—C10—H10B	108.7
C4—C3—C2	103.2 (3)	N1—C11—C12	125.0 (3)
C4—C3—H3A	111.1	N1—C11—C16	122.0 (3)
C2—C3—H3A	111.1	C12—C11—C16	113.0 (3)
C4—C3—H3B	111.1	C13—C12—C11	123.4 (3)
C2—C3—H3B	111.1	C13—C12—N2	114.5 (3)
H3A—C3—H3B	109.1	C11—C12—N2	121.5 (3)
C3—C4—C5	103.0 (3)	C14—C13—C12	119.2 (3)
C3—C4—H4A	111.2	C14—C13—H13	120.4
C5—C4—H4A	111.2	C12—C13—H13	120.4
C3—C4—H4B	111.2	C13—C14—C15	121.4 (3)
C5—C4—H4B	111.2	C13—C14—N3	118.8 (3)
H4A—C4—H4B	109.1	C15—C14—N3	119.7 (3)
N1—C5—C4	102.5 (3)	C16—C15—C14	117.9 (3)
N1—C5—H5A	111.3	C16—C15—H15	121.0
C4—C5—H5A	111.3	C14—C15—H15	121.0
N1—C5—H5B	111.3	C15—C16—C11	124.6 (3)
C4—C5—H5B	111.3	C15—C16—N4	116.2 (3)
H5A—C5—H5B	109.2	C11—C16—N4	119.1 (3)
N7—C6—C10	103.9 (3)	O7—C17—N7	121.3 (4)
N7—C6—C2	110.8 (3)	O7—C17—C18	122.6 (4)
C10—C6—C2	115.8 (3)	N7—C17—C18	116.1 (4)
N7—C6—H6	108.7	C17—C18—H18A	109.5
C10—C6—H6	108.7	C17—C18—H18B	109.5
C2—C6—H6	108.7	H18A—C18—H18B	109.5
C17—N7—C8	124.8 (3)	C17—C18—H18C	109.5
C17—N7—C6	119.9 (3)	H18A—C18—H18C	109.5
C8—N7—C6	113.0 (3)	H18B—C18—H18C	109.5
N7—C8—C9	104.2 (3)	O1—N2—O2	123.6 (3)
N7—C8—H8A	110.9	O1—N2—C12	118.3 (3)
C9—C8—H8A	110.9	O2—N2—C12	117.8 (3)
N7—C8—H8B	110.9	O4—N3—O3	123.6 (3)
C9—C8—H8B	110.9	O4—N3—C14	118.9 (3)
H8A—C8—H8B	108.9	O3—N3—C14	117.5 (3)
C8—C9—C10	106.0 (3)	O5—N4—O6	124.9 (3)
C8—C9—H9A	110.5	O5—N4—C16	117.5 (3)
C10—C9—H9A	110.5	O6—N4—C16	117.5 (3)
			
C11—N1—C2—C3	−175.5 (3)	N1—C11—C12—N2	18.7 (5)
C5—N1—C2—C3	−8.9 (3)	C16—C11—C12—N2	−162.5 (3)
C11—N1—C2—C6	58.6 (4)	C11—C12—C13—C14	−4.3 (5)
C5—N1—C2—C6	−134.7 (3)	N2—C12—C13—C14	166.1 (3)
N1—C2—C3—C4	30.8 (3)	C12—C13—C14—C15	−2.0 (5)
C6—C2—C3—C4	152.1 (3)	C12—C13—C14—N3	−179.1 (3)
C2—C3—C4—C5	−41.6 (4)	C13—C14—C15—C16	4.4 (5)
C11—N1—C5—C4	150.3 (3)	N3—C14—C15—C16	−178.5 (3)
C2—N1—C5—C4	−16.5 (4)	C14—C15—C16—C11	−0.9 (5)
C3—C4—C5—N1	35.4 (4)	C14—C15—C16—N4	175.7 (3)
N1—C2—C6—N7	−174.9 (2)	N1—C11—C16—C15	174.2 (3)
C3—C2—C6—N7	67.9 (4)	C12—C11—C16—C15	−4.6 (5)
N1—C2—C6—C10	67.2 (4)	N1—C11—C16—N4	−2.3 (5)
C3—C2—C6—C10	−50.0 (4)	C12—C11—C16—N4	178.9 (3)
C10—C6—N7—C17	−160.1 (3)	C8—N7—C17—O7	−170.4 (4)
C2—C6—N7—C17	74.9 (4)	C6—N7—C17—O7	−8.8 (5)
C10—C6—N7—C8	3.5 (4)	C8—N7—C17—C18	8.8 (5)
C2—C6—N7—C8	−121.4 (3)	C6—N7—C17—C18	170.5 (3)
C17—N7—C8—C9	176.1 (3)	C13—C12—N2—O1	−146.0 (3)
C6—N7—C8—C9	13.3 (4)	C11—C12—N2—O1	24.5 (4)
N7—C8—C9—C10	−24.7 (4)	C13—C12—N2—O2	28.0 (4)
C8—C9—C10—C6	27.4 (4)	C11—C12—N2—O2	−161.5 (3)
N7—C6—C10—C9	−18.8 (4)	C13—C14—N3—O4	174.7 (3)
C2—C6—C10—C9	102.9 (4)	C15—C14—N3—O4	−2.5 (5)
C5—N1—C11—C12	−126.2 (3)	C13—C14—N3—O3	−5.9 (5)
C2—N1—C11—C12	39.0 (4)	C15—C14—N3—O3	176.9 (3)
C5—N1—C11—C16	55.2 (4)	C15—C16—N4—O5	−114.2 (3)
C2—N1—C11—C16	−139.6 (3)	C11—C16—N4—O5	62.5 (4)
N1—C11—C12—C13	−171.6 (3)	C15—C16—N4—O6	64.1 (4)
C16—C11—C12—C13	7.2 (4)	C11—C16—N4—O6	−119.1 (3)

### Hydrogen-bond geometry (Å, º)

**Table:**

*D*—H···*A*	*D*—H	H···*A*	*D*···*A*	*D*—H···*A*
C2—H2···O1	0.98	2.18	2.891 (4)	129
C5—H5*B*···O5	0.97	2.59	3.157 (5)	118
C2—H2···O7	0.98	2.50	3.079 (4)	118
C18—H18*C*···O2^i^	0.96	2.51	3.454 (5)	168

Symmetry code: (i) −*x*+2, *y*+1/2, −*z*+3/2.
